# How structural biology transformed studies of transcription regulation

**DOI:** 10.1016/j.jbc.2021.100741

**Published:** 2021-05-04

**Authors:** Cynthia Wolberger

**Affiliations:** Department of Biophysics and Biophysical Chemistry, The Johns Hopkins University School of Medicine, Baltimore, Maryland, USA

**Keywords:** DNA-binding proteins, protein structure, nucleic acid structure, transcription factor, DNA–protein interaction, gene regulation, CAP, catabolite activator protein, PDB, Protein Data Bank, TBP, TATA-binding protein

## Abstract

The past 4 decades have seen remarkable advances in our understanding of the structural basis of gene regulation. Technological advances in protein expression, nucleic acid synthesis, and structural biology made it possible to study the proteins that regulate transcription in the context of ever larger complexes containing proteins bound to DNA. This review, written on the occasion of the 50th anniversary of the founding of the Protein Data Bank focuses on the insights gained from structural studies of protein–DNA complexes and the role the PDB has played in driving this research. I cover highlights in the field, beginning with X-ray crystal structures of the first DNA-binding domains to be studied, through recent cryo-EM structures of transcription factor binding to nucleosomal DNA.

The publication in the early 1980s of the first crystal structures of DNA and of proteins that bind to specific DNA sequences (1, 2, 3) marked a turning point in structural biology. X-ray crystallography had already made a profound impact on biology and biochemistry (4), beginning with the first atomic models of hemoglobin (5) and myoglobin (6), to the first structures of enzymes (7), antibodies (8), and tRNA (9, 10). At the same time, the need for large amounts of material to grow crystals of sufficient size and quantity restricted the field to naturally abundant proteins. With the exception of tRNA, it was not possible to obtain the homogeneous samples of RNA or DNA needed for crystallization trials. The advent of molecular cloning and strategies for overexpressing proteins in bacteria, however, dramatically increased the number and types of proteins whose structures could be determined. The publication in the early 1980s of structures of *E. coli* catabolite activator protein (CAP) (2) and of the bacteriophage lambda Cro (1) and *cI* (3) repressor proteins was electrifying and provided the first glimpses of how proteins might bind DNA and regulate transcription. The much broader set of biological problems to which structural methods could now be applied greatly increased the interest in structural biology. At the same time, the development of chemical methods to synthesize DNA oligonucleotides of defined length and sequence made it possible to crystallize and determine structures of DNA (11, 12), as well as of protein–DNA complexes. Indeed, it was the 1981 publication of the crystal structure of a B-DNA dodecamer by Dickerson and colleagues (1BNA) (12) that finally provided experimental proof for the B-DNA model proposed by Watson and Crick in 1953 (13). These combined developments in recombinant DNA technology and chemical synthesis of DNA marked the beginning of a new era in studies of protein–DNA interactions and gene regulation.

The advances in cloning and oligonucleotide synthesis played an additional role in expanding the impact of structural biology beyond simply making it possible to determine structures of protein–DNA complexes. The development of approaches that utilized oligonucleotides to engineer specific amino acid substitutions into proteins (14) meant that one could use structural information to introduce mutations that could be then be used to test mechanistic hypotheses based on crystal structures. An early example was the test of a model for how the helix-turn-helix element (15), which had been identified in early structures of DNA-binding proteins, mediated contacts with DNA base pairs. Site-directed mutagenesis of the bacteriophage 434 repressor validated the proposed model for DNA binding and provided clues as to how side chain contacts determined DNA sequence recognition (16). These new approaches that made it possible to use structural information to drive biochemical and genetic studies further broadened interest in structural biology and helped fuel a dramatic expansion in what had once been a relatively small community of X-ray crystallographers and NMR spectroscopists.

The ability to utilize the new structural information on DNA–protein complexes was, however, limited because many of these new structures were not broadly available. Although the Protein Data Bank (PDB) had been established more than a decade earlier, coordinate deposition was voluntary and many structures of proteins and oligonucleotides were not publicly available (17). Indeed, coordinates for the first DNA-binding proteins mentioned above, CAP (2), lambda cro (1), and lambda *cI* (3), were not deposited in the PDB. Recommendations from the International Union of Crystallography (18) and policy changes at the National Institutes of Health (19) and other funding entities led to mandatory coordinate deposition, making these exciting structures available to all investigators. The number and complexity of protein–nucleic acid complex structures have increased by many orders of magnitude since that time, fueled by technical advances in X-ray crystallography, nuclear magnetic resonance (NMR) spectroscopy and, most recently, cryo-electron microscopy (cryo-EM). The availability in the PDB of so many structures of individual transcription factors, enzymes, and nucleosomes has greatly facilitated structure determination of large complexes that contained many of these macromolecules. Most importantly, these structures are easily accessible to all outside the structural biology community and continue to drive new science.

This review focuses on the insights into the regulation of transcription gained from structural studies of protein–DNA complexes and the role the PDB has played in driving this research. I present a historical view of some of the milestones, beginning with structural studies of bacterial and phage repressor proteins bound to DNA, through structures of larger complexes determined by cryo-EM. I have provided the PDB ID in either the text or figure legend for each structure mentioned. Alas, a number of early structures were never deposited in the PDB, so in these cases I also provide a reference to a subsequent structure, along with its corresponding PDB ID. Given its focus on regulation, this review focuses on sequence-specific DNA-binding proteins and does not cover the structural studies of RNA polymerase or of the many transcription factors and chromatin-modifying enzymes required for transcription initiation and elongation. The reader is referred to several recent reviews that cover the remarkable structures of the eukaryotic (20, 21) and bacterial (22) transcription machinery.

## Recognition of specific DNA sequences

**Figure 1:**
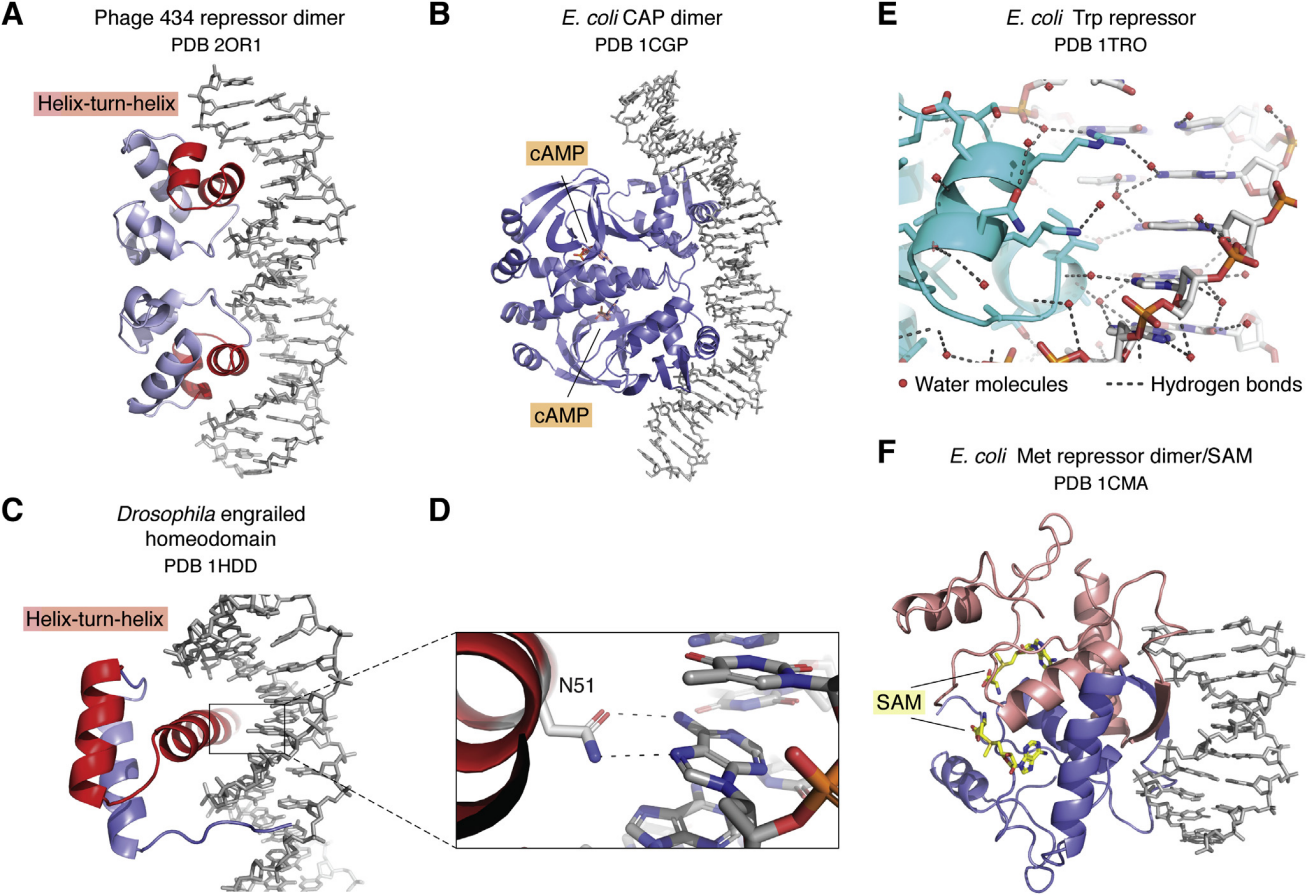
**Structures of bacterial and phage repressors and activators bound to DNA.***A*, phage 434 repressor dimer (2OR1) (28). The helix–turn–helix is highlighted in *red*. *B*, *E. coli* CAP dimer with cAMP bound to each monomer (1CGP). *C*, *Drosophila* engrailed homeodomain with helix–turn–helix is highlighted in *red* (1HDD). *D*, hydrogen bonding between Asn and Adenine (1HDD). *E*, *E. coli* Trp repressor showing waters (*spheres*) and water-mediated hydrogen bonds (1TRO). *F*, Met repressor dimer with two chains colored differently. There is one molecule of S-adenosine methionine (SAM) bound to each monomer (1CMA).

Regulation of specific genes depends on proteins that can recognize a particular sequence of DNA base pairs in a regulatory region. In bacteria, these proteins either activate or repress transcription by directly interacting with RNA polymerase (23). In eukaryotes, transcriptional regulators have separate domains that may recruit coactivator or corepressor complexes that attach or remove posttranslational modifications from histone, reposition nucleosomes, or promote assembly of the transcription preinitiation complex (24). Just a few years after structures of the first isolated DNA-binding domains mentioned above were elucidated (1, 2, 3), the first protein–DNA complexes reported in the mid-1980s marked the beginning in our understanding of the molecular basis for recognition of specific DNA sequences. Structures of complexes with the bacteriophage lambda (1LMB) (25, 26) and 434 repressors (2OR1) (27, 28) and cro (3CRO, 4CRO) (29, 30, 31) proteins showed how the second helix in the previously identified helix–turn–helix motif (15) inserted into the major groove of B-DNA (Fig. 1*A*). Side chains in the recognition helix contacted the edges of the DNA bases directly or *via* water-mediated hydrogen bonds, thereby contributing to sequence specificity, while other regions of the protein formed additional stabilizing contacts with the sugar–phosphate backbone. Although the bacteriophage repressors bound to relatively straight DNA, it turned out that the *E. coli* CAP protein (1CGP) induces a dramatic 90° bend in the helix axis (32) (Fig. 1*B*). This would be the first of many examples of proteins that induce bends and other distortions in the DNA that modulate the nature of sequence-specific contacts as well as (in most cases) increasing the buried surface area between protein and DNA. The helix-turn-helix motif was soon found in eukaryotic homeodomain proteins such as *Drosophila* engrailed (1HDD) (33) (Fig. 1, *C* and *D*) and yeast MATα2 (1APL) (34), although the longer recognition helix in homeodomains docked on DNA in a somewhat different manner. In general, all of these structures provided different examples of proteins that form chemically complementary interfaces with the DNA.

An unexpected twist on the nature of DNA sequence recognition emerged with the structure of the bacterial Trp repressor bound to DNA (35). Although Trp repressor also contains a helix–turn–helix, the protein forms no direct contacts with DNA bases. Instead, there are water-mediated contacts between Trp repressor and the base pairs in the major groove (Fig. 1*E*), with direct contacts formed only with the DNA backbone. The DNA sequence specificity of Trp repressor derives from sequence-dependent variations in DNA structure, a form of recognition termed indirect readout (35). Subsequent analyses have shown that sequence-dependent local variations in DNA structure play a role in a broad array of proteins that bind DNA (36).

**Figure 2:**
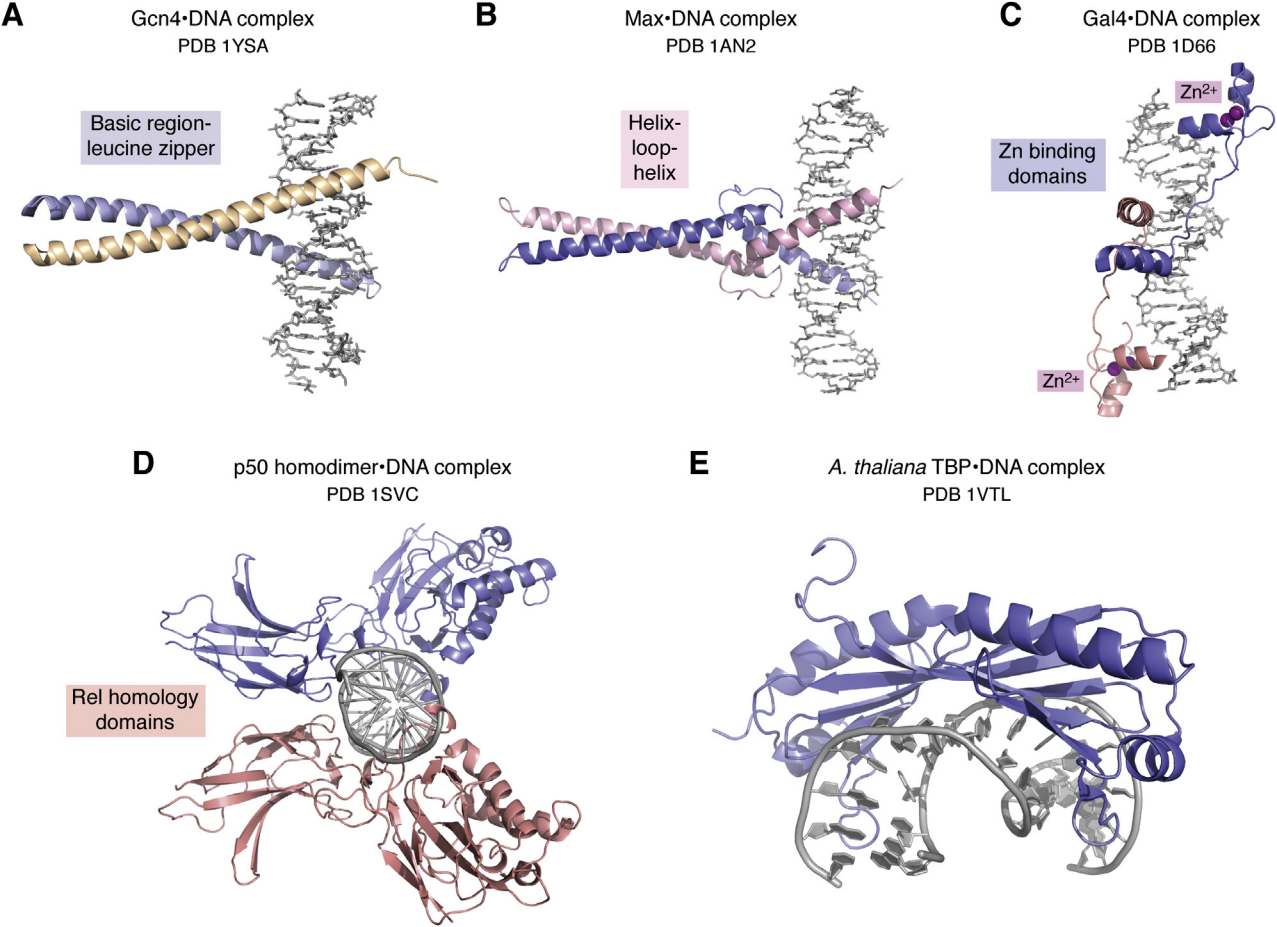
**Eukaryotic DNA-binding domains.***A*, Gcn4, a basic region-leucine zipper (bZIP) protein (1YSA). *B*, max, a helix–loop–helix protein (1AN2). *C*, Gal4. Each DNA-binding domain coordinates two Zn^2+^ ions (1D66). *D*, p50 homodimer, Rel-homology domain (1SVC). *E*, *A. thaliana* TBP bound to DNA (1VTL).

As more structures of complexes were determined, the remarkable structural diversity of sequence-specific DNA binding domains and the different modes of interaction with both the major and minor grooves quickly became evident. The early 1990s saw a veritable explosion in the number of novel DNA-binding domains. The structure of the DNA-bound bacterial Met repressor (1CMA) (37) revealed that a pair of beta strands fit in the major groove (Fig. 1*F*) just as well as an α helix. This validated a prediction, made well before any structures of DNA-binding proteins had been determined, that both α helices and β sheets had the optimal dimensions to fit in the major groove of B-DNA (38). Structures of eukaryotic transcriptional regulators such as the basic region-leucine zipper (bZIP) (39) (Fig. 2*A*), helix–loop–helix (40) (Fig. 2*B*), Gal4-type zinc binding domain (41) (Fig. 2*C*), and the immunoglobulin-like Rel homology domain (42, 43) (Fig. 2*D*) proteins represented yet other structurally distinct modes of docking on DNA and recognizing specific DNA sequences.

Perhaps the most unexpected finding from this era was the discovery of the dramatic DNA distortion induced by the eukaryotic TATA-binding protein (TBP), a subunit of the basal transcription factor complex, TFIID, that binds to the TATA box promoter element and helps nucleate assembly of the transcription preinitiation complex (44). In marked contrast to the proteins that insert helixes, strands, or loops into the DNA grooves, essentially forming a structurally complementary surface (see Figs. 1 and 2), it is the concave surface of TBP that contacts DNA in the minor groove (1YTB, 1VTL) (45, 46) (Fig. 2*E*). A severe distortion in the DNA, which contains a nearly 90° bend in the helix axis and is underwound, enables the concave surface of TBP to form sequence-specific contacts with bases in the minor groove.

**Figure 3:**
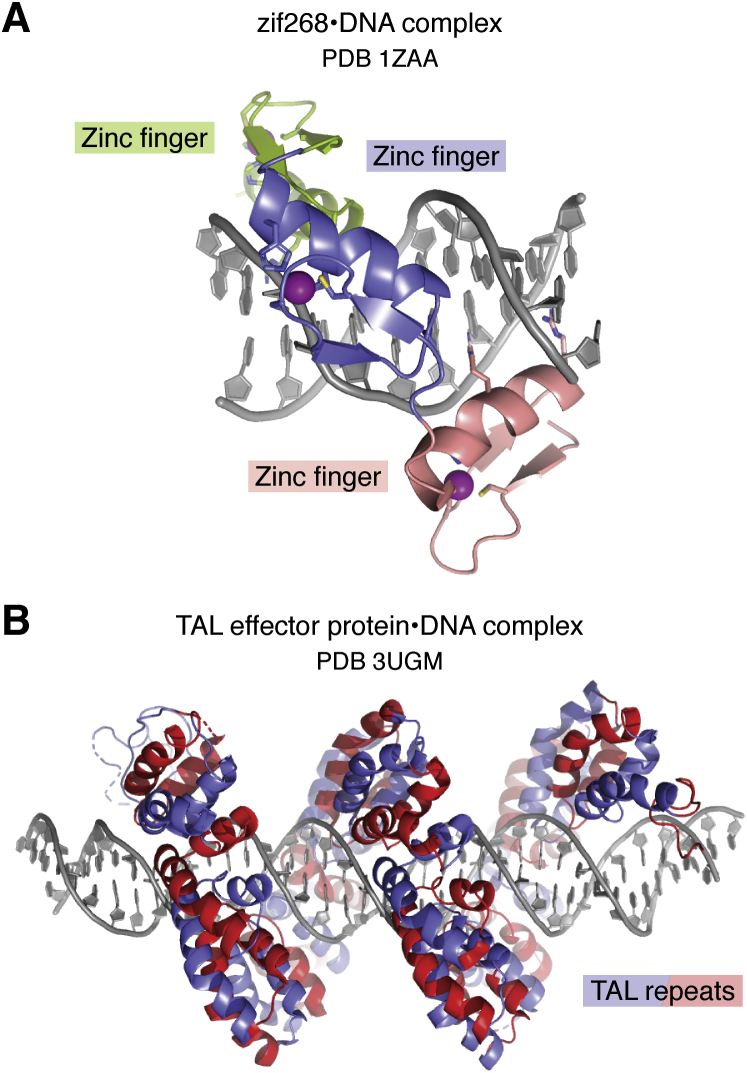
**Modular recognition of DNA sequence.***A*, zif268 has three zinc fingers (each colored differently) that bind DNA (1ZAA). Each zinc is coordinated by two His and two Cys side chains (*sticks*). Each finger contacts bases in the major groove with two side chains. *B*, TAL effector protein (3UGM). Alternate 34 amino acid TAL repeats are colored *red* and *blue*.

Zinc finger proteins were distinct from other classes of DNA-binding domains in their modular recognition of DNA sequences, and whose molecular details were first revealed in the structure of the three zinc fingers of Zif268 bound to DNA (1ZAA) (47) (Fig. 3*A*). Members of this large family of transcriptional regulators contain multiple tandem repeats of the ~33 amino acid domain with a structural zinc coordinated by two histidine and two cysteine side chains (48), with each zinc finger recognizing 3 to 4 base pairs (47) (Fig. 3*A*). The modular nature of zinc finger proteins presented an opportunity to engineer proteins with particular DNA-binding specificities (49, 50, 51, 52), which could then be used to target nucleases or other domains to specific sites in the genome (53, 54). This marked the first attempt at targeted genome engineering, which was followed a decade later by designed TAL effector proteins (55). Each repeat in these plant DNA-binding proteins recognizes a single base pair (56) (Fig. 3*B*), which greatly facilitated design of proteins with the desired DNA sequence specificity (57, 58) that could similarly be linked to endonuclease domains for genome engineering (55).

An analysis of PDB depositions as of the year 2000 (59) identified seven broad classes of sequence-specific DNA-binding proteins, with variations within each class. One of the common themes to emerge from all of these studies was the prevalence of DNA sequence recognition *via* contacts in the major groove, where the pattern of nucleobase functional groups is unique to each DNA sequence. Although it had initially been thought by some that there might be a recognition code in the form of a one-to-one correspondence between a particular base and one or more unique side chains, it became apparent early on that there was no such code (60), with the exception of the TAL effector proteins (57, 58). A comprehensive review of the determinants of DNA sequence recognition can be found in (61).

## Combinatorial regulation of transcription

**Figure 4:**
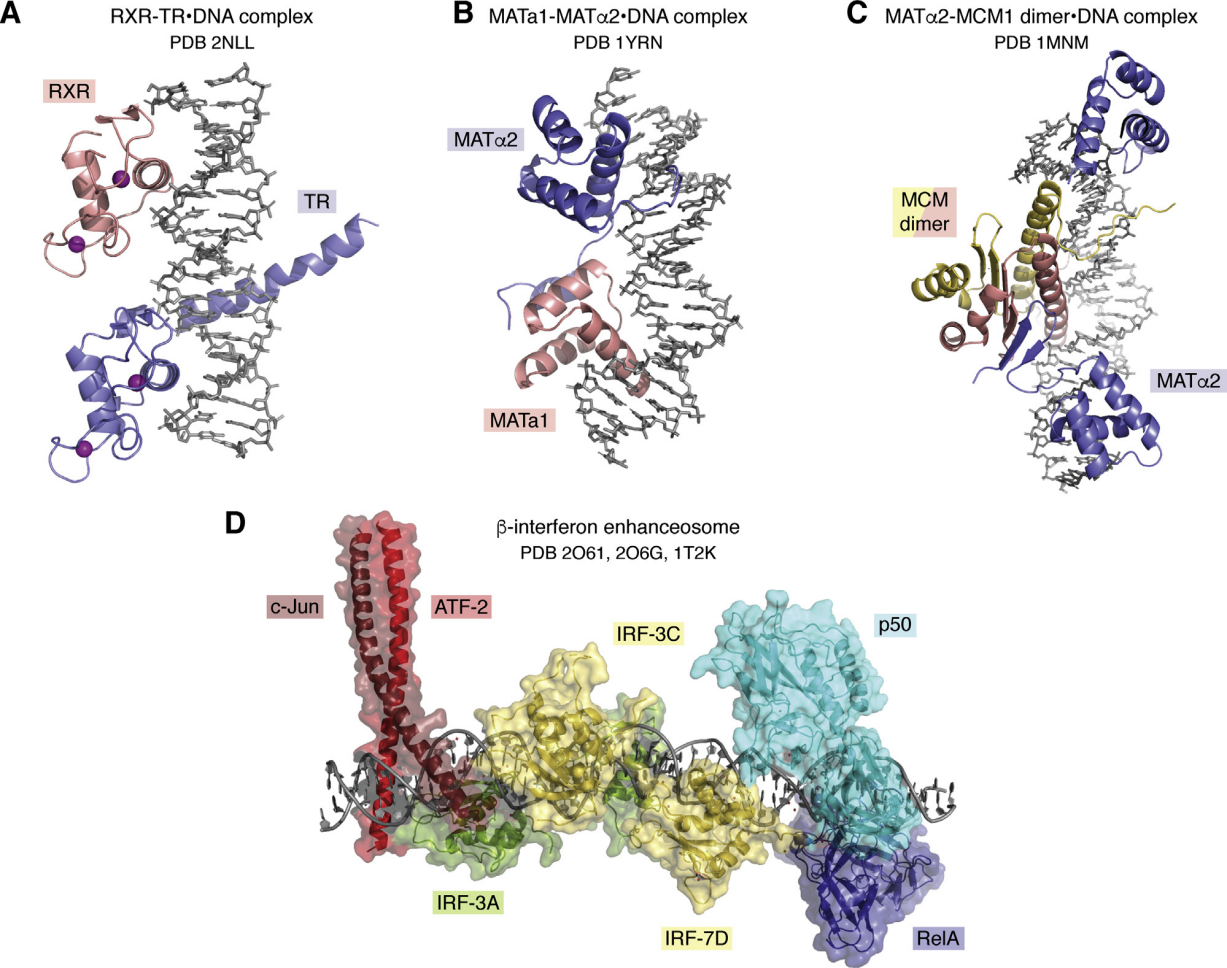
**Combinatorial regulation by multiprotein complexes.***A*, RXR (*salmon*)-TR (*blue*) (2NLL). *B*, Mat a1 (*salmon*)-Matα2 (*blue*) (1YRN). *C*, Matα2 (*blue*) and an MCM1 dimer (*salmon* and *yellow*) (1MNM). *D*, β-interferon enhanceosome. Model assembled from 2O61, 2O6G, 1T2K.

Many eukaryotic genes are regulated by multimeric complexes that can regulate transcription in response to multiple inputs. Beginning in the mid-1990s, structural studies of transcriptional regulators advanced to the next level of complexity, with structures determined of multiprotein complexes bound to DNA. One of the first was of the nuclear hormone receptor heterodimer composed of 9-*cis*-retinoic acid receptor (RXR) and thyroid hormone receptor (TR) (2NLL) (62) (Fig. 4*A*). Members of this family of DNA-binding proteins can form homodimers or heterodimers and contain separate ligand-binding domains, which change conformation upon ligand binding and recruit enzyme complexes that activate (coactivators) or repress (corepressors) transcription (63). Of interest, the RXR and TR DNA-binding domains bind DNA in tandem (62), in contrast with other members of this family, such as glucocorticoid receptor, which bind as symmetric dimers (64). Structural studies of the homeodomain superfamily revealed an even greater degree of complexity, as selected members of this family can heterodimerize with other homeodomain proteins or with DNA-binding proteins belonging to completely dissimilar structural families. The yeast MATα2 homeodomain protein, for example, can heterodimerize with a second homeodomain protein, MATa1 (1YRN) (65), or with MCM1 (1MNM) (66), a MADS box DNA-binding protein that is unrelated in structure to homeodomains (Fig. 4, *B* and *C*). Structures of *Drosophila* Ubx/Exd (1B8I) (67) and human HoxB1/Pbx1 (1B72) (68) homeodomain heterodimers bound to DNA provided additional insights into how transcription programs are regulated during development.

Through the late 1990s and early 2000s, structures of even larger complexes were determined. With the availability of many structures of smaller protein–DNA complexes in the PDB, it became possible to model regulatory regions and enhancers to which multiple proteins bind. The β-interferon enhancer, for example, contains binding sites within the 55-base pair enhancer sequence for eight proteins that bind cooperatively, together forming an “enhanceosome” (69). By combining structural information from several multiprotein subcomplexes (2O61, 2O6G, 1T2K), it was possible to assemble a model of the entire enhanceosome containing the bHLH proteins, C-Jun and ATF-2; the Rel homology domain proteins, p50 and RelA; and four IRF proteins, IRF-3A, 3C, 7B, and 7B (Fig. 4*D*) (70, 71).

## Transcription factor binding and chromatin

**Figure 5:**
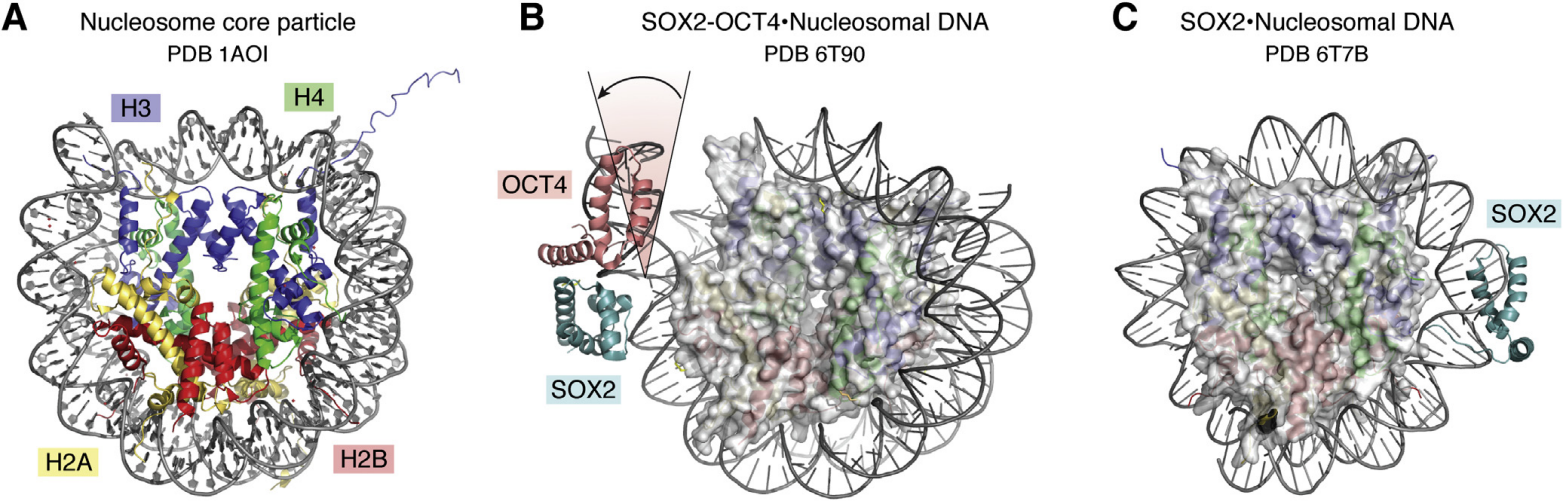
**Transcription factor binding to nucleosomal DNA.***A*, nucleosome core particle. Histones H2A (*yellow*), H2B (*red*), H3 (*blue*), H4 (*green*) (1AOI). *B*, Sox2 (*blue*) and Oct4 (*salmon*) bound to nucleosomal DNA near the exit site (1T90). *Lines* and *arrow* indicate the change in the path of the DNA as compared with the nucleosome in (*A*). *C*, Sox2 (*blue*) bound to an internal site of nucleosomal DNA (6T7B).

The packaging of eukaryotic DNA into chromatin impacts all cellular processes requiring access to DNA, including transcription. It is perhaps not surprising, then, that the structure of the fundamental organizational unit of the genome, the nucleosome (1AOI), is the most highly cited structure in the PDB (72). The first high-resolution structure of the nucleosome core particle, determined in 1997 (73), revealed the molecular details of how the 146-base pair DNA duplex wraps twice around the histone octamer core, which contains two copies each of histones H2A, H2B, H3, and H4 (Fig. 5*A*). One of the important observations to emerge from this and subsequent studies was that the DNA is not smoothly bent but instead contains local kinks that are favored by particular DNA sequences (74). These sequence-dependent differences in the relative energetic penalty for DNA kinking and bending thus play a key role in positioning nucleosomes at particular locations in the genome (74).

Most transcriptional regulators bind to nucleosome-depleted or nucleosome-free regions, where their DNA-binding domains can freely access the DNA. A class of regulatory proteins known as pioneer transcription factors, however, bind directly to nucleosomal DNA in regions of compacted chromatin and reprogram cell fate by altering local chromatin structure (75). Two recent cryo-EM structures have provided the first insights into how pioneer transcription factors, OCT4 and SOX2, bind to DNA that is simultaneously wrapped around the nucleosome (76, 77). The position of the recognition sequence within the nucleosomal DNA, or even whether there is a fixed site, is not clear, so the two studies inserted the recognition sequence in different locations in the DNA based on solution experiments. The structure of both OCT4 and SOX2 bound to DNA near the exit site from the nucleosome (superhelical location -6, or SHL-6) show the DNA peeled away from the histone core (Fig. 5*B*), suggesting a mechanism by which these pioneer factors could help open chromatin (6T90) (77). In structures of SOX2 (6T7B) and of a homolog, SOX11 (6T7A), bound to an internal DNA site at SHL +2 (Fig. 5*C*), the DNA is somewhat distorted and bulges away from the histone core (76). Together, these structures constitute an important start in understanding the mechanism by which these and other pioneer transcription factors open chromatin and alter transcription programs.

## Visualizing entire transcription initiation complexes

A more complete understanding of how DNA-binding proteins orchestrate transcription will require structures of ever-larger complexes containing all necessary components for transcription initiation. Thanks to the recent “resolution revolution” in cryo-EM (78), one structure after another of huge protein–nucleic acid complexes have provided unprecedented insights into transcription. Since virtually all of these complexes contain proteins whose structures had been determined, usually by X-ray crystallography, the ready availability of coordinates and associated data in the PDB has greatly facilitated map interpretation and model building. The many structures of basal transcription factors bound to DNA, such as TBP (1YTB, 1VTL) (45, 46), TFIIA (79), and TFIIB (80), and RNA polymerase II have provided the foundation on which to interpret structures of transcription initiation and elongation complexes (for a comprehensive recent review, see (21)). The change in the PDB coordinate data format to mmCIF/PDBx (81) was another advance that facilitated working with such large structures. The original PDB format, which was based on the number of characters that could fit on an IBM punch card, could only accommodate structures with up to 62 chains and fewer than 100,000 atoms. The mmCIF/PDBx format has no such limit and also accommodates additional metadata containing information about the macromolecules as well as experimental details. Thus, structures of a 2.7-MDa bacterial expressosome containing RNA polymerase, a ribosome, the NusG transcription factor, duplex DNA, mRNA, and tRNA could be accommodated in a single coordinate file, even though it contains 65 unique chains and more than 175,000 non-hydrogen atoms (6ZTJ, 6VU3) (82, 83). The rate at which these huge structures are emerging is so rapid that new structures of the RNA polymerase II preinitiation complex (2.6 MDa) appeared as this article was being revised (84).

## The future

What advances in studies of the mechanisms underlying transcription regulation can we anticipate in the near future and how will the PDB continue to support them? As structures have become more complex, it is increasingly common for multiple methods to be used to arrive at the final model. In addition to combining information from X-ray crystallography, NMR, cryo-EM and solution X-ray scattering, data from other biophysical and biochemical methods that provide complementary information are starting to be used to interpret cryo-EM maps of very large complexes with multiple components. This approach, referred to as integrative structural biology (85), incorporates information from methods such as mass spectrometry–cross-linking, hydrogen–deuterium exchange, Förster resonance energy transfer, chromosome capture, and many others. The opportunities presented by integrative structure determination have also presented challenges to the PDB as to how the data should be archived and displayed and how the models should be validated. The PDB has ongoing efforts to address these issues (86).

An important gap in our understanding of how transcription is regulated stems from our inability to capture the dynamics and time-dependent events that link structural snapshots. It is now possible to capture multiple states in a single cryo-EM sample, but linking them temporally involves extrapolation and educated guesswork. Solution and solid-state NMR, along with development of methods for time-resolved structure determination that can be applied to large assemblies, could help fill this gap. There is also the hope that it will eventually be possible to study all these events in their natural, cellular context with further development of cryo-electron tomography (87) or other imaging techniques that have yet to be invented. With so many possibilities ahead, now is as exciting a time in structural biology as when those first few structures of DNA-binding proteins were published 40 years ago.

## Conflict of interest

The author is a member of the scientific advisory board of Thermo Fisher Scientific.
